# Job vacancy at the European Centre for Disease Prevention and Control (ECDC)

**DOI:** 10.2807/1560-7917.ES.2023.28.8.2302231

**Published:** 2023-02-23

**Authors:** Eurosurveillance editorial team

**Affiliations:** 1 European Centre for Disease Prevention and Control (ECDC), Stockholm, Sweden

The European Centre for Disease Prevention and Control (ECDC) invites applications for the following position:

 

**Scientific Officer Communicable Diseases Prevention and Control**
The application deadline expires on 20 Mar 2023.

 

For more information, please visit the ECDC website: https://www.ecdc.europa.eu/en/vacancies

